# Resolutions Passed by the American Dental Association at the Last Meeting

**Published:** 1895-12

**Authors:** 


### Resolutions Passed by the American Dental Association at the Last Meeting.

(1.) That this association believes the conferring of honorary degrees in dentistry to be detrimental to the profession of dentistry, and hereby expresses its disapprobation of the practice.

(2.) That this association formally adopts the Army Medical Museum and Library as the National Museum and Library of the dental profession of the United States.

(3.) That a committee of five be appointed by the chair to co-operate with the Army Medical Museum and Library Managers in enriching its stores of dental literature and museum specimens, especially by appealing to dental societies and individual members of the dental profession for material assistance.

The committee appointed consists of Drs. Wm. Donnally, Washington, D. C.; J. Taft, Cincinnati, O.; H. J. McKellops, St. Louis, Mo.; Frank Abbott, New York City; and Henry W. Morgan, Nashville, Tenn.

(4.) That the American Dental Association condemns the use of secret preparations known as "local anæsthetics," as well as all other secret preparations.

(5.) That it is the sense of this association that the National Association of Dental Faculties can largely control the formation of dental colleges by passing a resolution refusing to admit any college to their association of which the organizers did not first apply and obtain permission from the National Association of Dental Faculties to organize such institutions.

(6.) That at the session of the American Dental Association to be held in 1896, the general sessions shall be held every morning and at such other times as may, at the pleasure of the association, be designated;

That the sections shall be required to hold separate meetings simultaneously during the day, and that all papers pertaining to the sections shall be read and discussed in the sections;

That at the *general sessions* one hour each session shall be devoted to the president's address, the general addresses prepared by appointment, and such other papers as embrace the results of original investigations as may be recommended by the appropriate section;

That at the general sessions reports embracing only a syllabus shall be made of the papers which shall have been accepted at the individual sections, these reports to be presented by the representative of said section: meaning hereby that the work must be done in the sections;

That all papers which have been accepted by the several sections shall be published in the transactions of the association, with the discussions thereon.

